# SIRT1 Mediates Melatonin’s Effects on Microglial Activation in Hypoxia: In Vitro and In Vivo Evidence

**DOI:** 10.3390/biom10030364

**Published:** 2020-02-27

**Authors:** Sara Merlo, Juan Pablo Luaces, Simona Federica Spampinato, Nicolas Toro-Urrego, Grazia Ilaria Caruso, Fabio D’Amico, Francisco Capani, Maria Angela Sortino

**Affiliations:** 1 Department of Biomedical and Biotechnological Sciences, Section of Pharmacology, University of Catania, 95123 Catania, Italy; sara_merlo@hotmail.com (S.M.); simona_spampinato@hotmail.com (S.F.S.); grazia.caruso@outlook.it (G.I.C.); 2 Laboratorio de Citoarquitectura y Plasticidad, Instituto de Investigaciones Cardiológicas, Universidad de Buenos Aires, Consejo Nacional de Investigaciones Científicas y Técnicas, Buenos Aires C1122, Argentina; juanpluaces@yahoo.com (J.P.L.); nicolas.toro3@gmail.com (N.T.-U.); franciscocapani@hotmail.com (F.C.); 3 Department of Biomedical and Biotechnological Sciences, University of Catania, 95123 Catania, Italy; f.damico@unict.it

**Keywords:** cobalt chloride, rat common carotid artery occlusion (CCAO), 5-methoxy-*N*-acetyltryptamine, melatonin receptors, silent mating type information regulation 2 homolog 1 (SIRT1), amoeboid microglia, nuclear factor-kappa B (NF-kB)

## Abstract

Melatonin exerts direct neuroprotection against cerebral hypoxic damage, but the mechanisms of its action on microglia have been less characterized. Using both in vitro and in vivo models of hypoxia, we here focused on the role played by silent mating type information regulation 2 homolog 1 (SIRT1) in melatonin’s effects on microglia. Viability of rat primary microglia or microglial BV2 cells and SH-SY5Y neurons was significantly reduced after chemical hypoxia with CoCl_2_ (250 μM for 24 h). Melatonin (1 μM) significantly attenuated CoCl_2_ toxicity on microglia, an effect prevented by selective SIRT1 inhibitor EX527 (5 μM) and AMP-activated protein kinase (AMPK) inhibitor BML-275 (2 μM). CoCl_2_ did not modify SIRT1 expression, but prevented nuclear localization, while melatonin appeared to restore it. CoCl_2_ induced nuclear localization of hypoxia-inducible factor-1α (HIF-1α) and nuclear factor-kappa B (NF-kB), an effect contrasted by melatonin in an EX527-dependent fashion. Treatment of microglia with melatonin attenuated potentiation of neurotoxicity. Common carotid occlusion was performed in p7 rats, followed by intraperitoneal injection of melatonin (10 mg/kg). After 24 h, the number of Iba1+ microglia in the hippocampus of hypoxic rats was significantly increased, an effect not prevented by melatonin. At this time, SIRT1 was only detectable in the amoeboid, Iba1+ microglial population selectively localized in the corpus callosum. In these cells, nuclear localization of SIRT1 was significantly lower in hypoxic animals, an effect prevented by melatonin. NF-kB showed an opposite expression pattern, where nuclear localization in Iba1+ cells was significantly higher in hypoxic, but not in melatonin-treated animals. Our findings provide new evidence for a direct effect of melatonin on hypoxic microglia through SIRT1, which appears as a potential pharmacological target against hypoxic-derived neuronal damage.

## 1. Introduction

Melatonin (5-methoxy-*N*-acetyltryptamine) is an endogenous neurohormone produced primarily by the pineal gland and mainly involved in the regulation of circadian rhythms. Aside from its classical action on sleep/wake cycles, melatonin has largely been shown to be a pleiotropic molecule [1,2,3,4] with multiple beneficial actions. In agreement, animal studies have shown melatonin to be an effective neuroprotectant in a number of neurodegenerative conditions such as hypoxia/ischemia [5,6,7], Alzheimer’s Disease [8], Parkinson’s Disease [9], and spinal cord injury [10]. Notably, clinical trials have been designed to establish the potential neuroprotective efficacy of melatonin in humans [11].

Melatonin selectively activates two G-protein coupled receptors, MT1 (or MTNR1a) and MT2 (or MTNR1b), which differ in tissue distribution, molecular structure, and downstream pathways [12,13,14,15]. The MT1 isoform, in particular, has been linked to neuroprotective actions of melatonin in different models [16,17,18] and appears to be the prevalent site of action of melatonin in human fetal brains [17]. 

Recent evidence linked melatonin’s neuroprotective effects to regulation of silent mating type information regulation 2 homolog (SIRT) 1 protein, a nicotinamide adenine dinucleotide (NAD)+-dependent class III histone deacetylase [19,20,21]. Acting through the deacetylation of histones as well as several non-histonic targets, SIRT1 is implicated in neuroprotection and longevity [22,23]. Melatonin appears to regulate SIRT1 expression through the MT1 receptor [21].

Cerebral hypoxia is among the pathological conditions for which melatonin has proven especially effective as a neuroprotectant. Particularly relevant is the beneficial potential of melatonin in perinatal hypoxia. Melatonin has been previously used in association with the standard hypothermic therapy in newborns affected by hypoxia, with positive results [24,25,26].

In the context of hypoxia, microglial activation plays a primary role, which appears to be dual. On one side, restorative responses are exerted via debris clearance and removal of excitatory terminals from injured neurons, while, on the other, pro-inflammatory responses contribute to the exacerbation of neuronal injury [27,28]. Particularly interesting is the presence in the corpus callosum (CC) of a population of amoeboid-shaped, active microglia, which prevail early during brain development, later switching to a more ramified phenotype [29]. These microglia are suggested to be mainly involved in the physiological clearance of cellular debris and the developmental shaping of axonal connectivity [29,30]. Notably, in the last few years, the CC was shown to be an early and more common target of hypoxic damage than previously understood [31].

We have recently suggested that microglia may represent a feasible target for early intervention in different neuroinflammatory conditions [32,33,34]. Importantly, melatonin was shown to inhibit microglial pro-inflammatory polarization in hypoxic conditions in animal models [35,36,37].

Based on these premises, the aim of the present work was to explore the involvement of SIRT1 in the effects of melatonin on microglial activation early after induction of hypoxia. To this end, we used both in vitro and in vivo approaches. In vitro, chemical hypoxia was induced with cobalt chloride (CoCl_2_) in rat primary microglia, BV2 murine microglial cell line, and differentiated neuronal-like cell line SH-SY5Y. In vivo, hypoxia was obtained by common carotid artery occlusion (CCAO) in p7 rats, followed by induction of anoxia. Melatonin was administered intraperitoneally at the end of hypoxia. Our results show that melatonin protects microglia from the hypoxic insult and attenuates its pro-inflammatory polarization, providing for the first time, evidence for a link between these effects and SIRT1 activation in microglia. In addition, our in vitro data show that melatonin’s action on microglia leads to an indirect beneficial action on neuronal survival. 

## 2. Materials and Methods 

### 2.1. Drugs and Reagents

CoCl_2_ was purchased from Sigma-Aldrich (St. Louis, MO, USA) as a 100 mM stock solution. Melatonin, EX527 (Santa Cruz Biotechnologies, Santa Cruz, CA, USA) and BML-275 (Enzo Life Sciences, Inc., Farmingdale, NY, USA) were dissolved in dimethyl sulfoxide (DMSO) as 10 mM stocks and further diluted in culture medium for experiments. 

### 2.2. Cell Cultures

Mixed glial cultures were prepared from 1–3 days-old Sprague-Dawley rats (Harlan Laboratories, Indianapolis, IN, USA) according to an established protocol in our lab [33]. Briefly, the cortex was dissected, meninges removed, and tissue trypsinized (Invitrogen, Carlsbad, CA, USA) to a single cell suspension and filtered through a 40 μm nylon cell strainer (BD Biosciences, Erembodegem, Belgium). Cells were plated onto 75 cm^2^-flasks and maintained in Dulbecco’s Modified Eagle Medium (DMEM) supplemented with 10% fetal bovine serum (FBS), penicillin (100 U/mL)/streptomycin (100 µg/mL) at 37 °C and CO2 atmosphere. For microglial isolation, after 8–10 d, confluent mixed cultures were shaken at 250 rpm at 37 °C for 1.5 h to collect microglia. For experiments, microglia or mixed glia were plated in 35 mm-dishes at a density of 2.5 million and 1 million cells, respectively. All glial cells were used for the experiments 48 h after re-plating.

BV2 mouse microglial cell line (ATTC, LGC Standards, Manassas, VA, USA) was grown in RPMI medium supplemented with 10% FBS at 37 °C and CO2 atmosphere. SH-SY5Y human neuroblastoma cells (ATTC) were grown in DMEM/F12 1:1 medium supplemented with 10% FBS at 37 °C and CO2 atmosphere. Cells were differentiated by gradual serum deprivation and overnight starvation prior to treatments, as previously published by our group [33].

All medium constituents were obtained from Invitrogen. All experimental animal procedures were carried out in accordance with the directives of the Italian and European Union regulations for the care and use of experimental animals (DL116/92) and were approved by the Italian Ministry of Health.

### 2.3. Animals

Sprague Dawley pregnant rats were obtained from the central vivarium at the School of Veterinary Sciences, University of Buenos Aires. Rats were maintained at 21 ± 2 °C and 65 ± 5% humidity with free access to food and water, under a 12:12 h light/dark cycle (lights on 7:00 a.m.). Each animal was used only once, and all efforts were made to minimize the suffering of the animals and to reduce the number of animals used. All procedures were performed in accordance with the Argentina National Institute of Health Guide for the Care and Use of Laboratory Animals (Animal Welfare Assurance, A-3033-01/protocol#S01084) and were previously approved by the Ethics Committee at the University of Buenos Aires (CICUAL#4091/04).

### 2.4. Model for Common Carotid Artery Occlusion (CCAO) and Treatment

The model for CCAO used in this study has been previously developed and validated [38,39]. P7 male Sprague-Dawley rats were anesthetized (40 mg/kg ketamine and 4 mg/kg xylazine) and placed on a heat plate to keep their body temperature at constant 37 °C. The right common carotid artery (CCA) was exposed through an incision on the neck and was then isolated and permanently ligated with a 6-0 surgical silk thread (hypoxia-ischemia; HI group *n* = 13). The wound was then closed, and the pups were returned to their dams for 4–5 h to recover. Subsequently, the animals were subjected to a 100% nitrogen environment at 37 °C for 3 min to induce anoxia. Sham-operated rats (sham group *n* = 12) had their right CCA exposed but not ligated, and no nitrogen was applied. One hour after nitrogen exposure, animals were injected intraperitoneally (i.p.) with vehicle solution (vehicle group *n* = 12) or with 10 mg/kg of melatonin (melatonin group *n* = 13). At 24 h anoxia (postnatal day 8), animals were sacrificed, and their brains were collected for further analysis. Melatonin (Sigma) was dissolved in DMSO and diluted in normal saline to a final concentration of 5% DMSO.

### 2.5. Viability 3-(4, 5-Dimethylthiazolyl-2)-2, 5-Diphenyltetrazolium Bromide (MTT) Assay

One hour before the end of treatments, MTT (0.5 mg/mL; Sigma) was added to the medium and cells further incubated for 1 h at 37 °C. The medium was removed, and cell lysis was carried out by incubation with DMSO for 10 min at 37 °C. The absorbance of solubilized formazan crystals was measured at 545 nm with VarioskanTM Flash Multimodal Reader (Thermofisher Scientific, Waltham, MA, USA).

### 2.6. Trypan Blue Exclusion Assay

Cells were stained with 0.4% Trypan blue solution for 10 min and washed with PBS. The number of blue-stained, Trypan-permeable dead cells vs. the total number of cells were counted in five random fields/well under phase-contrast microscopy.

### 2.7. Real-Time Polymerase Chain Reaction

Cells were collected, and total RNA extracted using the RNeasy Plus Mini Kit (Qiagen, Milan, Italy). The RNA concentration was determined using Nanodrop spectrophotometer ND-1000 (Thermofisher), and 2 μg of RNA were reverse transcribed using Superscript-VILO kit (Invitrogen) according to the manufacturer’s instructions. Quantitative real-time PCR was performed from 100 ng/sample of cDNA with Rotor-Gene Q using Qiagen QuantiNova SYBR Green Real Time-PCR Kit. Primers are listed in Table 1. Melting curve analysis confirmed the specificity of the amplified products. Data were analyzed applying the ΔΔCt method and expressed as a fold change vs. control.

### 2.8. Western Blot

Cells were collected and lysed in M-PER^®^ Mammalian Protein Extraction Reagent (Thermofisher Scientific) supplemented with anti-protease and anti-phosphatase cocktails (Sigma). Rat hippocampal and cortical tissues were isolated and lysed in radioimmunoprecipitation assay buffer (RIPA) buffer with anti-protease and anti-phosphatase cocktails (Sigma). Samples were sonicated, centrifuged at high speed for five minutes at 4 °C and protein concentration was determined by micro Bradford reagent (Sigma) protocol, according to the manufacturer’s instructions. Absorbance was measured with a Varioskan^TM^ Flash Multimode Reader. Sodium dodecyl sulphate-poly-acrylamide gel electrophoresis (SDS-PAGE) was performed by loading equal amounts of protein extracts/experiment on pre-cast 4-20% gradient gels (Bio-Rad, Hercules, CA, USA) and followed by transfer to nitrocellulose membrane (Hybond ECL, Amersham Biosciences Europe GmbH, Milan, Italy) using a Transblot semidry transfer cell (Bio-Rad). Membranes were blocked with Odyssey Blocking buffer and incubated with primary antibodies overnight at 4 °C. Primary antibodies used were: rabbit anti-MT1 (1:300; Thermofisher, Cat. No. PA5-75749), rabbit anti-SIRT1(H300) (1:400; Santa Cruz, Cat. No. sc-15404), rabbit anti-NF-kBp65 (1:400; Thermofisher, Cat. No. PA1-186), rabbit anti-β-actin (1:5000; Sigma, Cat. No. A2066) and mouse anti- glyceraldehyde 3-phosphate dehydrogenase (GAPDH) (1:5000; Millipore, Billerica, MA, USA, Cat. No. MAB374). Membranes were then washed and exposed to appropriate IRDye^®^ 680RD or IRDye^®^ 800CW secondary antibodies (1:15000; LI-COR Biosciences, Lincoln, NE, USA) or Alexa Fluor-conjugated secondary antibodies (1:5000; Thermofisher) for 45 min at room temperature (RT). The detection of specific bands was carried out using the LI-COR Odyssey^®^ Infrared Imaging System or the iBright FL1000 Imaging System. Band intensity was analyzed using the ImageJ software, developed by the National Institutes of Health (NIH) and in the public domain. All blots were cropped to display only specific bands of interest.

### 2.9. Immunocytochemistry

Fixation was carried out with ice-cold 4% paraformaldehyde (30 min), followed by permeabilization when necessary with 0.1% Triton X-100 on ice (10 min) and blocking in 3% bovine serum albumin (BSA;30 min). Incubation with primary antibodies was carried out in a 3% BSA solution (overnight) at 4 °C. Primary antibodies used were: rabbit anti-SIRT1(H300) (1:400; Santa Cruz, Cat. No. sc-15404), rabbit anti-NF-kB p65 (1:400; Thermofisher, Cat. No. PA1-186), mouse anti-HIF-1α(H1alpha-67) (1:80; Santa Cruz, Cat. No. sc-53546). After washing, cells were incubated with secondary antibodies for 45 min RT, washed, and mounted with 4′,6-diamidino-2-phenylindole (DAPI)-containing mounting solution (both from Sigma). Secondary antibodies used were: Alexa-Fluor 546-anti-mouse (1:300; Invitrogen), Alexa-Fluor 488-anti-rabbit (1:300; Invitrogen). Digital images were captured with a Zeiss Observer.Z1 microscope equipped with the Apotome.2 acquisition system (Zeiss, Oberkochen, Germany).

### 2.10. Immunohistochemistry and Cell Count

At P8, intracardiac perfusion was performed as described previously [40]. Animals were anesthetized with intraperitoneal administration of ketamine 40 mg/kg and xylazine 4 mg/kg, and perfused intracardially with 4% paraformaldehyde in 0.1 M phosphate buffer, pH 7.4. Brains were removed and post-fixed in the same fixative solution for 2 h at RT and then immersed overnight at 4 °C in 0.1 M phosphate buffer, pH 7.4. Then, the brains were dehydrated through an ascending ethanol series and separately included in paraffin wax.

Coronal whole brain sections (5 μm thickness) were cut with the RM2235 microtome (Leica, Wetzlar, Germany) and mounted onto salinized slides. Heat-induced antigen retrieval was performed in 10 mM sodium citrate (Sigma) and 0.05% Tween 20, pH 6. Non-specific labeling was blocked using 10% normal fetal bovine serum (FBS) for 30 min at RT. Double (simultaneous) immunofluorescence staining was performed by incubating tissue sections with the mixtures of primary antibodies: rabbit polyclonal anti-Iba1-AIF1 (1:700; Novus Biologicals, Centennial, CO, USA, Cat. No. NBP2-19019) and mouse monoclonal anti SIRT1(B7) (1:200; Santa Cruz, Cat. No. sc-74465), mouse monoclonal anti-Iba1(GT10312) (1:200; Thermofisher, Cat. No. MA5-27726) and rabbit monoclonal anti-phospho-NF-κB p65 (Ser536) (93H1) (1:1200; Cell Signaling Technology, Danvers, MA, USA, Cat. No. #3033). After washing in PBS+0.1%Tween (PBS-T), sections were simultaneously incubated for 30 min at RT with secondary antibodies (all from Thermofisher): goat Alexa-Fluor 488-conjugated anti-rabbit (1:300) + donkey Alexa-Fluor 546-conjugated anti-mouse (1:250). After washing in PBS-T, slides were mounted with DAPI-containing Fluoromount mounting medium (Sigma).

The number of Iba1 immunoreactive cells in different brain areas was determined in two slides/brain and at least eight counting frames per animal. For each photo, cells were counted in two squares of 200 µm by 200 µm. The number of Iba1+ cells with nuclear SIRT1 localization in the CC was determined in two slides/brain and at least six counting frames per animal. 

### 2.11. Statistical Analysis

All in vitro data were from at least three independent experiments run at least in triplicate. For primary microglia, experiments were from separate isolations. In vivo experiments were carried out on at least three animals/group. All experimental values are presented as the mean ± SEM. Statistical analyses were performed, as appropriate, by Student’s t-test or one-way ANOVA followed by Neuman–Keuls post-hoc test using GraphPad Prism Software (GraphPad Software, San Diego, CA, USA). *P* < 0.05 was the criterion for statistical significance.

## 3. Results

### 3.1. In Vitro Experiments

#### 3.1.1. Microglia Potentiate Neuronal Damage during Chemical Hypoxia with CoCl_2_

**Figure 1:**
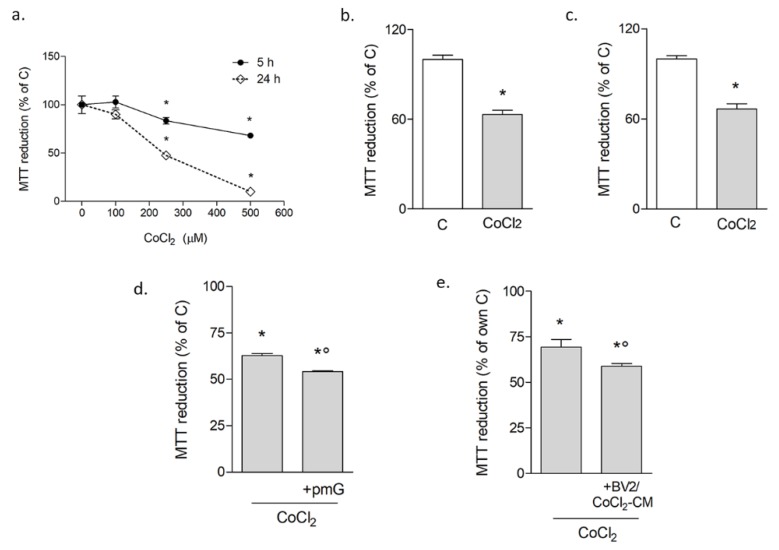
CoCl_2_ reduces cell viability in neuronal-like and microglial cells. The time and concentration-response curve for CoCl_2_ in SH-SY5Y neuronal-like cells, tested by the MTT assay (**a**). Effects of CoCl_2_ (250 μM for 24 h) on primary microglia (**b**) and BV2 cell line (**c**). CoCl_2_ toxicity on SH-SY5Y in co-culture with primary microglia (+pMG; **d**) or treated in conditioned medium (+CM) from CoCl_2_-pulsed BV2 cells (**e**). Bars are mean ± SEM of at least three independent experiments. * *p* < 0.05 vs. own zero (**a**) or vs. respective control (**c**) and ° *p* < 0.05 vs. other groups by one-way ANOVA followed by Newman–Keuls test for statistical significance.

CoCl_2_ toxicity was initially tested on differentiated neuronal-like cells SH-SY5Y. A concentration-response curve was carried out by the MTT assay, testing effects at early (5 h) and late (24 h) time points (Figure 1a). The results confirmed the time and concentration-dependent neurotoxic effects of CoCl_2_, which appeared significant starting at 250 μM. Based on these data, this concentration was used in all subsequent experiments. The MTT assay on both primary rat microglial cells (Figure 1b) and BV2 cells (Figure 1c) showed significant reduction of survival after 24 h of exposure to CoCl_2_. CoCl_2_ toxicity was confirmed by trypan blue exclusion assay both on SH-SY5Y cells (viable cells: CTR 84.71% ± 1.08 vs. CoCl_2_ 58.38% ± 3.39) and BV2 cells (viable cells: CTR 96± 0.65 vs. CoCl_2_ 69.47 ± 3.74). To explore whether and how microglia affected neuronal susceptibility to hypoxia-induced injury, neuronal-like cells were co-cultured with primary microglia plated on transwell inserts. In the presence of microglia, CoCl_2_ resulted slightly but significantly in more toxicity, as revealed by the MTT assay (Figure 1d). A similar effect was observed when neuronal-like cells were exposed to conditioned medium (CM) from BV2 cells pulsed with CoCl_2_ for 3 h, followed by an 18 h-recovery to obtain CM devoid of residual CoCl_2_ (Figure 1e).

#### 3.1.2. Melatonin Protects Microglia after Hypoxia via SIRT1 Activation

**Figure 2:**
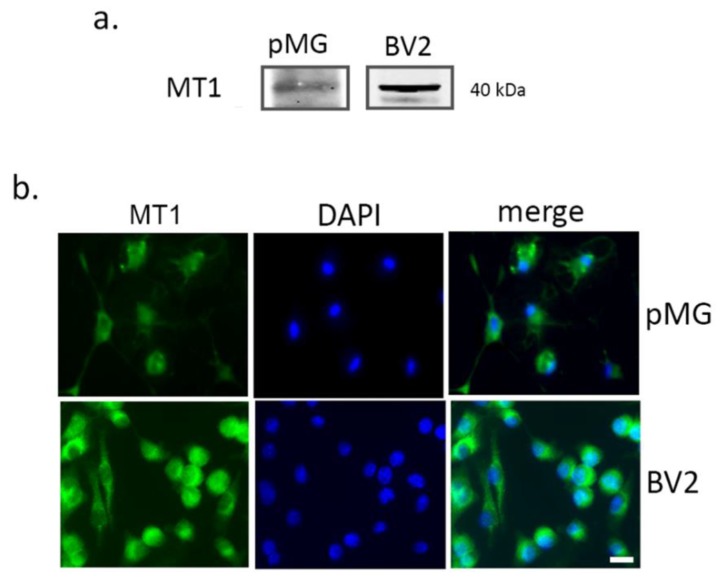
Melatonin receptor 1 (MT1) is expressed on microglial cells. Western blot (**a**) and immunocytochemical analysis (**b**) of the MT1 receptor in primary microglia (pMG) and BV2 cell line. Representative images are shown. Scale bar = 20 μm.

The expression of the selective MT1 receptor in microglia was tested prior to investigating the effects of melatonin. Western blot analysis was carried out to ascertain the specificity of the antibody used (Figure 2a) and immunocytochemical analysis (Figure 2b) confirmed receptor expression in both primary microglia and BV2 cells.

**Figure 3:**
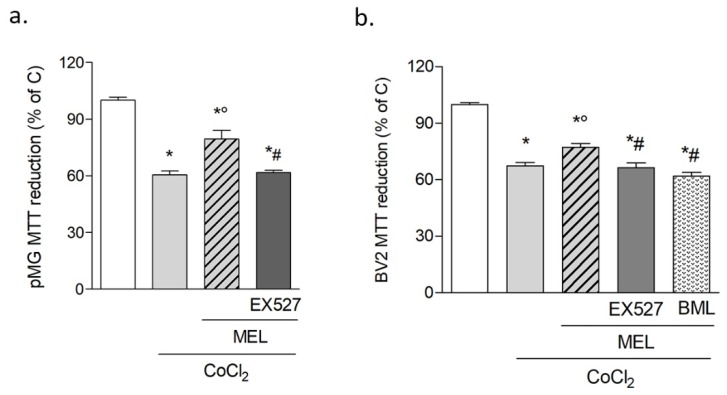
Melatonin protects microglia against CoCl_2_ toxicity via SIRT1. CoCl_2_ (250 μM for 24 h) toxicity on primary microglia (**a**) and BV2 cells (**b**) in the presence of melatonin alone (1 μM) or in combination with SIRT1 inhibitor EX527 (5 μM) or BML-275 (2 μM), assessed by the MTT assay. Bars are mean ± SEM of at least three independent experiments. * *p* < 0.05 vs. respective control (C), ° *p* < 0.05 vs. CoCl_2_ and #*p* < CoCl_2_ + melatonin (MEL) by one-way ANOVA followed by Newman–Keuls test for statistical significance.

In addition, the effects of melatonin on CoCl_2_–induced inflammatory challenges were preliminarily determined. To this end, mRNA expression of interleukin (IL)-6, IL-1β, and tumor necrosis factor (TNF)-α was analyzed by real-time quantitative PCR after 5 h of exposure to CoCl_2_. The results confirmed that CoCl_2_ increased cytokine transcription (5.23 ± 1.27; 3.64 ± 0.9 and 4.5 ± 1.44-fold change vs. C for IL-6, IL-1β, and TNF-α, respectively). These effects were prevented by melatonin (0.76 ± 0.14; 0.44 ± 0.04 and 1.18 ± 0.21-fold change vs. C for IL-6, IL-1β, and TNF-α, respectively). Accordingly, the direct addition of 1 μM melatonin in combination with CoCl_2_ slightly but significantly increased survival of both primary microglia (Figure 3a) and BV2 cells (Figure 3b). The involvement of SIRT1 in melatonin’s action was addressed in these conditions by selective pharmacological inhibition with EX527 (5 μM), which was shown to prevent melatonin’s protective effects in both primary microglia (Figure 3a) and BV2 cells (Figure 3b). Additionally, blockade of AMP-activated protein kinase (AMPK) pathway, classically linked to SIRT1 activation, with selective inhibitor BML-275 (2 μM) abolished melatonin’s protective effects against CoCl_2_ (Figure 3b). Given the highly superimposable responses of primary microglia and BV2 cells, the latter were used for all further investigations. 

**Figure 4:**
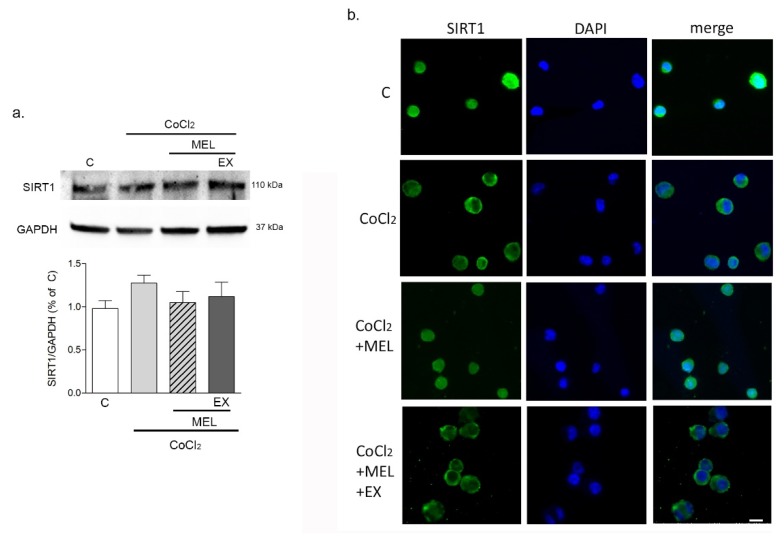
CoCl_2_ prevents SIRT1 nuclear translocation in microglia. In (**a**), Western blot analysis of SIRT1 expression in BV2 cells exposed to CoCl_2_ (250 μM for 8 h) alone, or in combination with either melatonin (1 μM) or melatonin + EX527 (5 μM). In (**b**), immunostaining of SIRT1 (green) and nuclear counterstaining with DAPI (blue) in BV2 cells treated with CoCl_2_ alone or in combination with melatonin (1 μM) or melatonin + EX527 (5 μM). Bars are mean ± SEM of three independent experiments. Representative images are shown. Scale bar = 20 μm.

The responses of BV2 cells were then analyzed at an early time point (8 h) with the intention to precede massive cell death. Analysis of SIRT1 expression by Western blot (Figure 4a) showed that SIRT1 protein levels remained unchanged early (8 h) after exposure of BV2 cells to either CoCl_2_ alone or in combination with melatonin (1 μM). However, the deacetylase’s subcellular localization appeared modified by CoCl_2_, as shown by immunocytochemical imaging (Figure 4b; green). SIRT1 was highly nuclear in control conditions, prevalently cytosolic after exposure to CoCl_2_, and restored into the nucleus by 1 μM melatonin. Notably, such an effect was precluded when SIRT1 was selectively inhibited with EX527 (5 μM). 

#### 3.1.3. Melatonin Modulates Microglial Hypoxic and Inflammatory Markers Indirectly Affecting Neurons during Hypoxia

**Figure 5:**
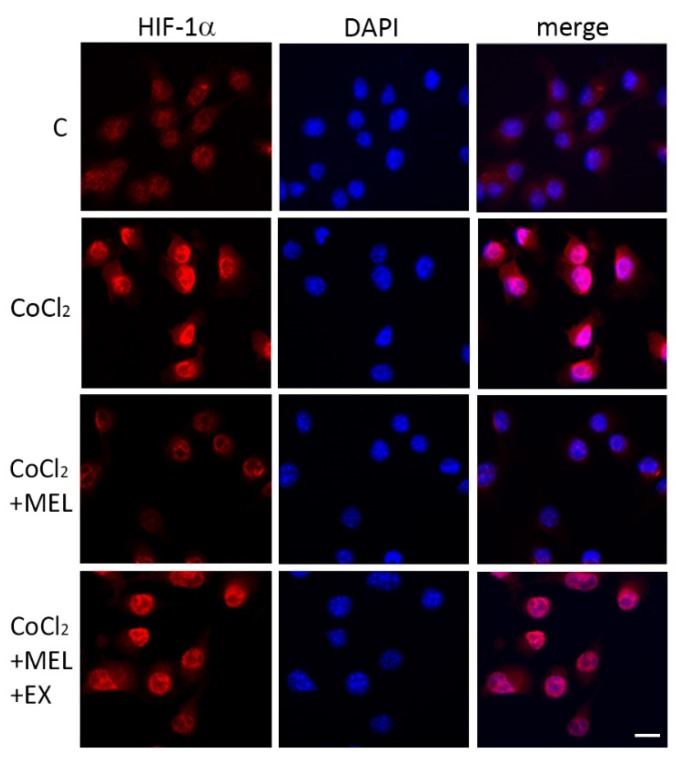
CoCl_2_ induces hypoxia-inducible factor (HIF)-1α in microglia. Immunostaining of HIF-1α (red) and nuclear counterstaining with DAPI (blue) in BV2 cells following exposure to CoCl_2_ alone (250 μM for 8 h) or in the presence of melatonin (1 μM) and melatonin + EX527 (5 μM). Representative images are shown. Scale bar = 20 μm.

The distinctive hypoxic marker HIF-1α was analyzed by immunostaining in BV2 cells exposed to CoCl_2_ for 8 h. Results showed that CoCl_2_ increased nuclear localization compared to the control, where only a faint signal was detectable (Figure 5). In the presence of melatonin (1 μM), nuclear HIF-1α was reduced, an effect that appeared sensitive to SIRT1 inhibition with EX527 (5 μM; Figure 5). 

**Figure 6:**
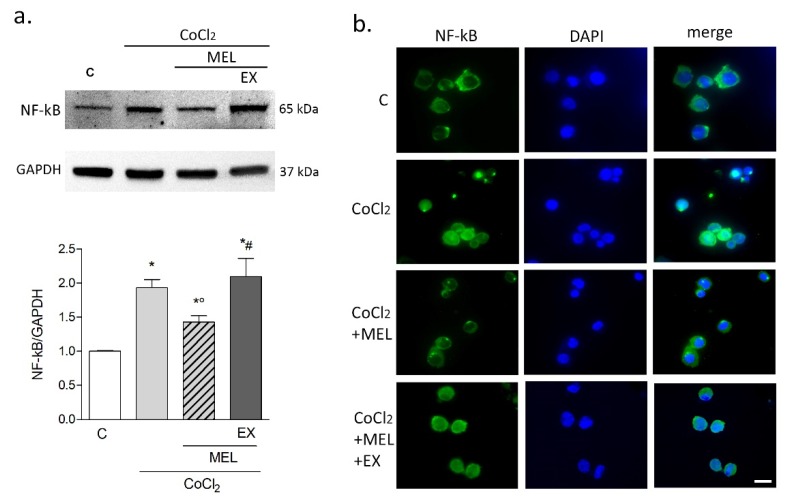
Melatonin attenuates NF-kBp65 upregulation by CoCl_2_ in microglia. Western blot analysis (**a**) of NF-kBp65 levels in BV2 cells exposed to CoCl_2_ (250 μM for 8 h) alone, or in combination with either melatonin (1 μM) or melatonin + EX527 (5 μM). In (**b**), immunostaining of NF-kBp65 (green) and nuclear counterstaining with DAPI (blue) following exposure to CoCl_2_ alone (250 μM for 8 h), or in the presence of melatonin (1 μM) + EX527 (5 μM). Bars are mean ± SEM of three independent experiments. * *p* < 0.05 vs. control (C), ° *p* < 0.05 vs. CoCl_2_ and #*p* < 0.05 vs. CoCl_2_ + MEL by one-way ANOVA followed by Newman–Keuls test for statistical significance. Representative images are shown. Scale bar = 20 μm.

Expression of the p65 subunit of the inflammatory marker NF-kB was analyzed by Western blot at the same time point (8 h). CoCl_2_ induced a significant increase in NF-kB expression compared to control. This effect was attenuated by melatonin but restored with the addition of EX527 (Figure 6a). Consistent with this result, immunostaining nuclear localization of NF-kBp65 was increased by CoCl_2_ exposure, and such effect prevented by melatonin in an EX527-sensitive manner (Figure 6b). 

**Figure 7:**
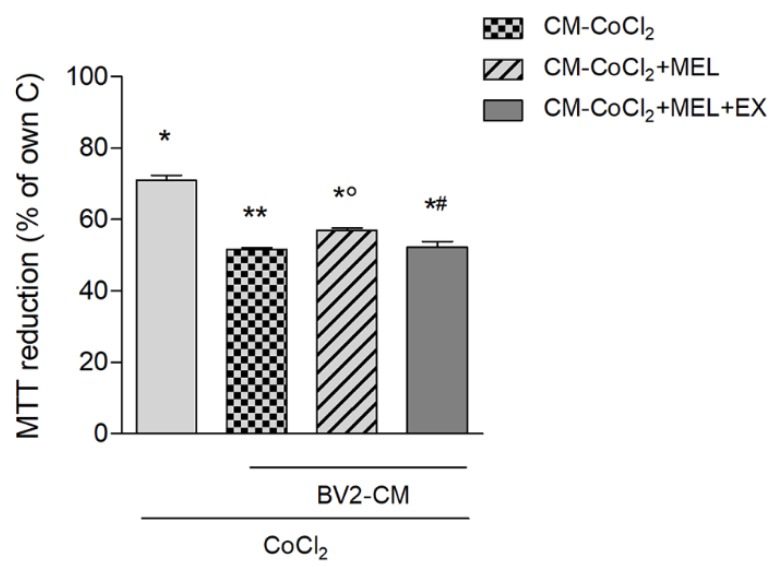
Melatonin modifies the microglial response to CoCl_2_, reducing its effects on hypoxic neuronal damage. BV2 cells were pulsed with CoCl_2_ (250 μM) alone or in combination with melatonin (1 μM) or melatonin+EX527 (5 μM) for 3 h, followed by washing and recovery in fresh medium devoid of any drug. Conditioned medium (BV2-CM) was collected after further 18 h of incubation and transferred to SH-SY5Y during challenge with CoCl_2_ (250 μM for 24 h). Neuronal-like cell viability was then tested by the MTT assay. Bars are mean ± SEM of three independent experiments. * *p* < 0.05 vs. own control, ° *p* < 0.05 vs. CoCl_2_ + MEL + EX527. Another group by one-way ANOVA followed by Newman–Keuls test for statistical significance.

Finally, we assessed the indirect outcome of melatonin’s modulation of hypoxic microglia on neuronal vulnerability to hypoxia. To this end, a conditioned medium (CM) protocol was chosen where BV2 cells were pulsed with CoCl_2_, alone or in combination with drugs, and recovered in fresh medium devoid of drugs and thus enriched only with released factors (see the Materials and Methods section for detailed protocol). Results showed that exposure of neuronal-like cells to CoCl_2_ (24 h) in the presence of CM, collected from BV2 microglia pre-pulsed with CoCl_2_ (BV2-CM- CoCl_2_), exacerbated neuronal death at the MTT assay (Figure 7). Such potentiation of neuronal damage was mitigated in the presence of CM from BV2 cells pre-treated with CoCl_2_+melatonin (BV2-CM- CoCl_2_+MEL; Figure 6). Finally, CM derived from BV2 pre-pulsed with CoCl_2_ + melatonin + EX527 (BV2-CM- CoCl_2_ + MEL + EX) restored full toxicity (Figure 7).

### 3.2. In Vivo Experiments

#### 3.2.1. Melatonin Differently Affects Microglia in the Cortex and Hippocampus of Hypoxic Rats

**Figure 8:**
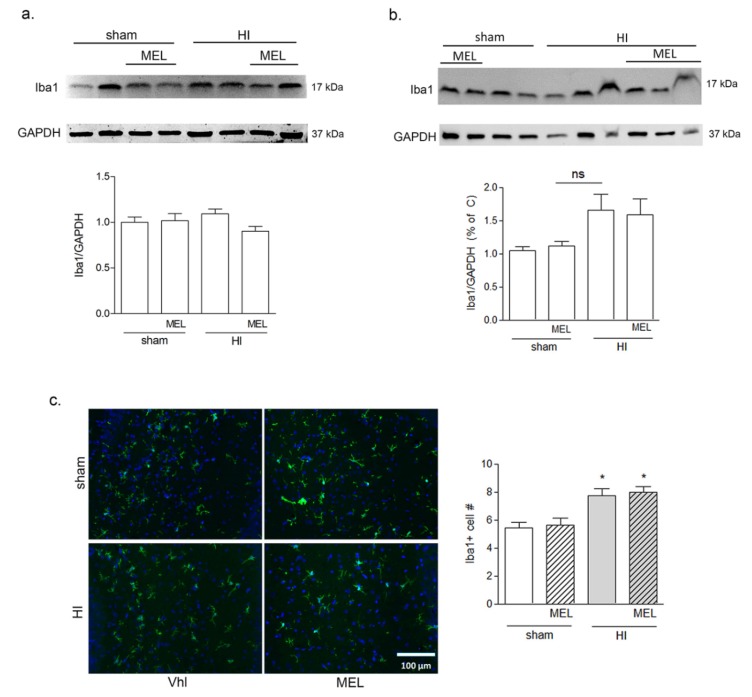
Melatonin does not affect Iba1+ cell number in rats subjected to CCAO. Seven-day-old rats were subjected to sham surgery or to ligation of the right carotid artery followed by hypoxia. Melatonin-treated groups were subsequently injected with melatonin (10 mg/kg) and sacrificed after 24 h. Iba1 expression in ipsilateral cortical (**a**) and hippocampal (**b**) protein extracts was evaluated by Western blot analysis. Microglia were labeled by immunohistochemical staining of Iba1 (green, **c**) with DAPI nuclear counterstaining (blue, **c**). Double positive Iba1+/DAPI+ cells were counted in the CA1 area of the hippocampus (HC; graph in **c**). Bars are mean ± SEM of at least three animals/group. * *p* < 0.05 vs. sham by one-way ANOVA followed by Newman–Keuls test for statistical significance. Representative images of blots and of ipsilateral HC are shown. Scale bar = 100 μm.

Seven-day old rats were subjected to common carotid artery occlusion followed by hypoxia, as described in detail in the Materials and Methods section. Melatonin (10 mg/kg) was injected i.p. after induction of anoxia, and animals sacrificed 24 h later. Expression of selective microglial marker Iba1 was analyzed by Western blot in ipsilateral hippocampal and cortical protein extracts of sham-operated or hypoxic (HI) animals, treated or not with melatonin (MEL). The results showed no effects on Iba1 expression in the cortex (Figure 8a), while a trend of the increase was evident in the hippocampus, although it did not reach statistical significance (Figure 8b). Immunostaining for Iba1 was carried out on whole-brain sections, and the number of microglial cells was determined by counting positive cells in the ipsilateral areas of interest. Microglia in the hippocampus appeared highly branched, and their number increased significantly in hypoxic animals compared to sham-operated groups (Figure 8c). Melatonin did not appear to modify such an increase (Figure 8c). Interestingly, a small population of microglial cells displaying an activated amoeboid phenotype was evident selectively in the CC. Their number did not significantly differ between treatment groups (not shown).

#### 3.2.2. Expression of SIRT1 Is Selectively Modified in Amoeboid Microglia of the Corpus Callosum in Hypoxic Rats

**Figure 9:**
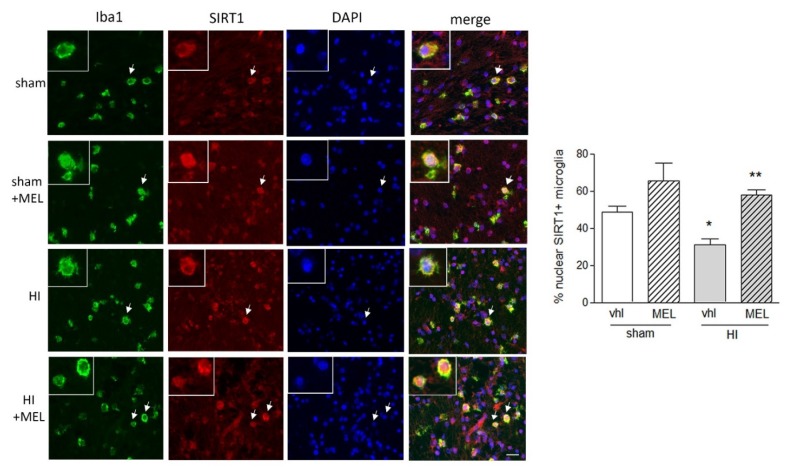
Melatonin promotes nuclear SIRT1 localization in amoeboid microglia of the corpus callosum in rats subjected to CCAO. Seven-day-old rats were subjected to sham surgery or to ligation of the right carotid artery followed by hypoxia (HI). Melatonin (MEL)-treated groups were subsequently injected with melatonin (10 mg/kg) and sacrificed after 24 h. Double immunohistochemical staining of Iba1 (green) and SIRT1 (red) with DAPI nuclear counterstaining (blue) is shown. The graph reports the percentage of nuclear SIRT1+, Iba1+ microglial cells in the area. Representative images of ipsilateral CC are shown. Insets show magnification of cells indicated by the arrows. Scale bar = 50 μm. Bars are mean ± SEM of at least three animals/group. * *p* < 0.05 vs. sham and ** *p* < 0.05 vs. HI by one-way ANOVA followed by Newman–Keuls test for statistical significance.

Expression of SIRT1 in microglial cells was investigated by double immunostaining with Iba1 on whole brain sections. Microglia in the hippocampus and cortex did not appear to express clearly detectable levels of SIRT1. Remarkably, however, amoeboid microglia in the CC (green, Figure 9) appeared clearly positive for SIRT1 (red; Figure 9). Moreover, SIRT1 localization was prevalently cytoplasmic in the hypoxic group (HI), while it appeared with a more defined nuclear localization in melatonin-exposed animals, both sham and hypoxic (sham + MEL and HI + MEL; Figure 9). This was confirmed by counting the number of amoeboid Iba1+ cells displaying nuclear SIRT1, which was significantly lower in hypoxic animals but restored in the presence of melatonin (graph in Figure 9). 

**Figure 10:**
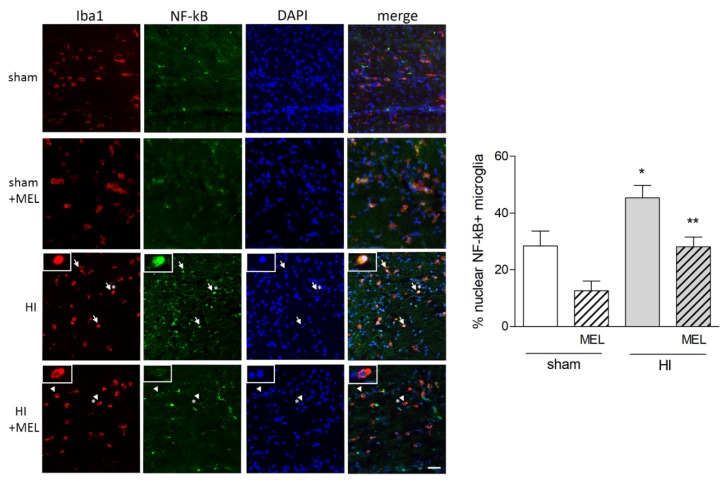
Melatonin antagonises nuclear NF-kB localization in amoeboid microglia of the corpus callosum in rats subjected to CCAO. Seven-day-old rats were subjected to sham surgery or to ligation of the right carotid artery followed by hypoxia (HI) alone or with subsequent injection of melatonin (10 mg/kg; HI + melatonin (MEL). Animals were sacrificed after 24 h. Double immunohistochemical staining of Iba1 (red) and NF-kB (green) with DAPI nuclear counterstaining (blue) is shown. The graph reports the percentage of nuclear SIRT1+, Iba1+ microglial cells in the area. Representative images of ipsilateral CC are shown. Arrows indicate representative Iba+ cells with nuclear NF-kB; arrowheads indicate representative Iba1+ cells with extranuclear NF-kB staining. Asterisks indicate cells reported in the insets at higher magnification. Scale bar = 40 μm. Bars are mean ± SEM of at least three animals/group. * *p* < 0.05 vs. sham and ** *p* < 0.05 vs. HI by one-way ANOVA followed by Newman–Keuls test for statistical significance.

Immunohistochemical analysis of pNF-kBp65 in the same area showed a defined nuclear localization in amoeboid microglia of hypoxic animals (HI), compared to both sham-operated and hypoxic melatonin-treated (sham and HI + MEL) groups (Figure 10). The number of NF-kB+/Iba1+ microglia was significantly increased in hypoxic animals compared to the other groups, together with the number of cells where it was localized in the nucleus. 

## 4. Discussion

Melatonin is an endogenous hormone characterized by neuroprotective activities, exerted through multiple mechanisms. Its production is regulated by light-dark cycles, with peaks of release during the dark period and suppression by light [1]. Melatonin has been largely shown to protect against hypoxic injury with the reduction of the infarct area [5,41,42]. Noteworthy, increased mortality following hypoxia-ischemia has been reported in MT1 receptor knockout mice [17]. Melatonin’s ability to easily cross the blood-brain barrier and its safety profile make it an ideal candidate, especially for pediatric use against long term neurological deficits that develop following prenatal/perinatal hypoxia. Hypoxic-ischemic encephalopathy is, in fact, a still frequent condition responsible for neonatal morbidity and mortality [26]. Brain or whole-body hypothermia is currently the only therapeutic option offering amelioration in the prognosis, as from preclinical and clinical data [26,43,44,45]. In addition, evidence of a benefit from adjunctive therapy with melatonin has been described [46,47], and a recent study on melatonin safety, pharmacokinetics (PK), and dosage in the neonatal population reported positive results [25]. Finally, it is worth noting that a significant increase of melatonin was described following hypoxia both in experimental models and in human stroke, suggesting even the existence of an endogenous protective response on its part [48]. The detailed comprehension of melatonin’s mechanisms of action is hence of paramount importance. So far, studies on the protective activity of the hormone in hypoxia have mainly focused on direct neuronal effects, whereas evidence regarding melatonin’s involvement in the modulation of inflammatory responses [49], as also shown by our data, prompted us to turn our attention to the effects on microglia that indirectly influence neurons. Microglia are, in fact early responders to hypoxic insults and can deeply affect neuronal vulnerability [50]. Furthermore, we aimed to explore the intriguing possibility that SIRT1, a deacetylase endowed with manifold protective actions and extensively studied in neurons [22], could be a mediator of melatonin’s effects on microglia against hypoxic insults. 

Melatonin has been reported to act both on selective G-protein coupled receptors and as an antioxidant. This latter function can be both direct, due to melatonin’s scavenger properties targeted in particular to mitochondria, or indirect through modulation of the expression of antioxidant enzymes [51,52,53]. In addition, melatonin has been shown to directly interact with target proteins modulating their function [4,54]. In our work, we first confirmed the expression of melatonin receptor MT1 in both primary and immortalized microglia. MT1 is coupled to Gi and Gq proteins, and its activation inhibits the formation of cyclic AMP, activates AMP-activated protein kinase (AMPK) signaling, and inhibits phospho-CREB and protein-kinase A signaling [55,56,57]. This isoform, in particular, was here chosen because of its known involvement in the neuroprotective actions of melatonin, as from experimental models of newborn hypoxic-ischemic brain injury [17]. In addition, MT1 was previously shown to mediate activation of SIRT1 in other tissues, such as the liver [58]. 

In the present study, we used a previously established in vitro model of chemical hypoxia with CoCl_2_, which stabilizes the α subunit of the transcription factor HIF-1, dampening its degradation [59]. The main cellular responses between cobalt and low oxygen-induced hypoxia have been reported to be significantly similar [60]. HIF-1 upregulation takes place strictly during hypoxia to activate selective response genes [61,62,63,64]. Interestingly, a cell-type-specific role has been suggested for HIF-1, whose upregulation in glial cells appears to trigger detrimental effects [65,66,67,68,69]. In agreement, in our model, nuclear HIF-1 was highly expressed in microglia following CoCl_2_ exposure, and microglial viability was significantly reduced. Previous in vitro studies have shown microglial cell death in CoCl_2_-induced hypoxic conditions [70,71]. In line with this negative role for HIF-1, its myeloid-specific knock-out in mice has been shown to reduce neuronal and microglial death following hypoxia, lowering inflammation, and improving behavioral recovery [72]. Likewise, the knockdown of HIF-1 was sufficient to elicit an anti-inflammatory effect in BV2 cells exposed to CoCl_2_ [72]. 

Melatonin was directly protective against microglial damage, and this effect proved to be dependent on SIRT1 and its downstream AMPK pathway [73,74,75], as shown by the use of selective pharmacological inhibitors. Interestingly, we did not detect any variation in SIRT1 levels, but rather found a shift between its cytoplasmic or nuclear localization in different conditions. While the detrimental effects exercised by hypoxia coincided with a cytoplasmic localization, melatonin promoted SIRT1 presence in the nucleus, both under basal and hypoxic conditions. Hence, the involvement of SIRT1 in our conditions is supported by its activation state, as here determined by analysis of its localization, together with the effects of pharmacological inhibition. Consistent with this notion, the subcellular localization of SIRT1 actually accounts for its differential actions: in particular, nuclear SIRT1 is endowed with protective anti-inflammatory action [76], while cytoplasm-localized SIRT1 was shown to enhance apoptosis in different cancer cell lines [77,78]. Despite previous reports indicating that SIRT1 levels declined following in vivo hypoxia and were restored by melatonin, this was observed selectively in neurons [21,79,80].

In order to determine if improvement of microglial survival induced by melatonin/SIRT1 was coupled with a direct action on the microglial inflammatory phenotype, we analyzed the effects of treatment on expression/localization of transcription factors HIF-1α and NF-kB. As previously described for HIF-1, NF-kB was also selectively detected in the nuclei in hypoxic conditions, consistent with its activation and polarization of microglia to sustain an incipient inflammatory reaction [81]. It is important to consider that both HIF-1 [82] and NF-kB [83] are direct targets of SIRT1, which keeps them inactivated by deacetylation. The antagonistic cross-talk between SIRT1 and both NF-kB and HIF-1 has been demonstrated to play a role in inflammation and energy metabolism; perturbations in this signaling can lead to chronic inflammation [84]. In light of this, the opposite compartmentalization we found for HIF-1 and NF-kB on one side and SIRT1, on the other, is perfectly in line with their predicted state of activation and downstream effects. 

In order to further assess the role of microglia as a target of melatonin action, we extended our investigation to an in vivo model of hypoxia. The permanent ligation of the common carotid artery represents a validated and widely used model of perinatal hypoxia. Postnatal 7-day rats were chosen since, at this time, the developmental stage is histologically similar to that of a 32- to 34-week gestation human fetus or newborn infant [85]. Aiming to analyze the effects of melatonin early during the development of cell damage, we chose a 24 h time-point previously shown to correspond to the initial phases of microglial activation, preceding full neuronal damage [86]. Among the different areas examined for a microglial response, only in the CA1 area of the hippocampus, we detected an increase in the number of microglia, an effect that, however, was not modified by melatonin. Iba1-positive cells of the hippocampus in our conditions did not show the typical amoeboid phenotype of activated microglia, but rather displayed long thin processes and did not express detectable levels of SIRT1. Modulation of hippocampal SIRT1 by melatonin has been previously described, but it was confined to the neuronal cell population [79]. On the contrary, intense staining for SIRT1 was evident in the population of amoeboid microglia of CC, characterized by round cell bodies devoid of ramifications. Even more remarkably, the enzyme was nuclear in melatonin-treated, but not in hypoxic animals. Amoeboid microglia of the CC have been previously described as a transient population of active microglia physiologically involved in the early developmental stages of periventricular white matter [87]. Our finding in the model of hypoxia appears even more compelling in light of the high vulnerability to hypoxic injury described, especially for this area, in the newborn [7,88]. Amoeboid microglia, in particular, has in fact been shown to be implicated in the early (present at 3 h following injury) inflammatory response induced by hypoxia in this area of the neonatal brain, leading to periventricular white matter and neuronal damage as wells as disruption of the immature blood-brain-barrier [88,89,90,91]. On these bases, we focused our attention on this microglial population to confirm the ability of melatonin to affect the inflammatory response as observed in the in vitro model. Accordingly, the prevalently nuclear localization of NF-kB in the hypoxic group was indicative of a state of inflammation, a condition that was counteracted by melatonin. Our data are in agreement with the suggested antagonistic crosstalk between NF-kB and SIRT1 [84] and suggest that the fine balance between the two factors may mediate the response of CC amoeboid microglia to melatonin to contrast the hypoxia-induced inflammatory phenotype. In line with this, recent evidence from a model of perinatal hypoxia suggested that microglial HIF-1, through miRNA-210, targets SIRT1, reducing NF-kB deacetylation, and rescuing its inflammatory activity [92].

## 5. Conclusions

In conclusion, we have shown in vitro that microglial SIRT1 plays a major role in response to melatonin following hypoxic injury and appears able to dampen the neuroinflammatory event. In vivo, at the early time point examined, this occured in a specific microglia subpopulation localized in the CC that is selectively vulnerable to hypoxia early during brain development. These findings overall confirm microglia as an additional target for the action of melatonin in hypoxia with a mechanism involving SIRT1.

## Figures and Tables

**Table 1:** Primers used for real-time polymerase chain reaction (PCR) amplification.

PRIMERS	Manufacturer
Mm_Il6_1_SG QuantiTect Primer Assay (mouse)–(IL-6)_QT00098875	Qiagen
Mm_Tnf_1_SG QuantiTect Primer Assay (mouse)–(TNF-α)_QT00104006	Qiagen
Mm_Il1b_2_SG QuantiTect Primer Assay (mouse)–(IL-1β)_QT01048355	Qiagen
Ribosomal Protein S18-Forward (GTTCCGACCATAAACGATGCC) Ribosomal Protein S18-Reverse (TGGTGGTGCCCCCGTCAAT)	Eurofin
